# Understanding the Feature Space and Decision Boundaries of Commercial WAFs Using Maximum Entropy in the Mean

**DOI:** 10.3390/e25111476

**Published:** 2023-10-24

**Authors:** Henryk Gzyl, Enrique ter Horst, Nathalie Peña-Garcia, Andres Torres

**Affiliations:** 1 Centro de Finanzas IESA, Caracas 1010, Venezuela; henryk.gzyl@iesa.edu.ve; 2 School of Management, Universidad de los Andes, Bogota 111711, Colombia; 3 Research Department, CESA Business School, Bogota 110311, Colombia

**Keywords:** cyber security, web application firewalls, feature space, decision boundary, ill-posed linear inverse problem, maximum entropy in the mean

## Abstract

The security of a network requires the correct identification and characterization of the attacks through its ports. This involves the follow-up of all the requests for access to the networks by all kinds of users. We consider the frequency of connections and the type of connections to a network, and determine their joint probability. This leads to the problem of determining a joint probability distribution from the knowledge of its marginals in the presence of errors of measurement. Mathematically, this consists of an ill-posed linear problem with convex constraints, which we solved by the method of maximum entropy in the mean. This procedure is flexible enough to accommodate errors in the data in a natural way. Also, the procedure is model-free and, hence, it does not require fitting unknown parameters.

## 1. Introduction

In the ever-changing digital environment, the dangers posed by cyber-attacks have become increasingly critical. The growing complexity of malicious actors, together with the swift proliferation of connected devices and systems, has resulted in an unparalleled degree of vulnerability for both individuals and organizations (see ISACA (https://www.isaca.org/go/state-of-cybersecurity-2021 (accessed on 23 September 2023))). Consequently, it is crucial to devise and employ innovative intrusion detection techniques that are capable of effectively combating these emerging threats.

Web application firewalls (WAFs) play a vital role in safeguarding contemporary applications, as the majority of attacks target the application layer of the OSI model [1]. Although commercial WAFs have exhibited superior overall performance in comparison to their open-source counterparts [2], they pose a challenge for defenders due to the absence of transparency concerning the feature space, learning algorithm, and decision function utilized by these systems. The feature space encompasses the set of all potential features that can be employed to characterize a data point, while decision boundaries represent the demarcations that distinguish different classes within the feature space. This knowledge gap obstructs defenders’ ability to optimize their usage of commercial WAFs and enhance their defenses against cyber-attacks. Furthermore, recent research has indicated that even widely used classification algorithms can be circumvented with a high likelihood by attackers, even if the attacker has access to only a small surrogate dataset of the classifier and limited knowledge of the targeted system [3,4]. Research has made significant advances when it comes to the automatic detection of attacks; most of the methodologies proposed are based on very complex models that require a lot of time in processing and computing resources [5]. One of the most prevalent trends is the use of feature selection algorithms to reduce the cost associated with the training and inference of these models. Some examples include employing filter-based feature reduction using the XGBoost algorithm [6], utilizing genetic algorithms (GAs) in conjunction with a logistic regression (LR) wrapper-based feature selection methodology [7], and implementing hybrid sampling with deep hierarchical networks [8], among others. Following this trend, our model-free approach is a tool that eases the amount of resources required to detect and prevent cybersecurity attacks and blusters the ability of defendants to differentiate between malicious and benign traffic, even in scenarios where there is limited access to information, as is the case with users of commercial firewalls. In other areas, researchers have approached modeling cooperation and extortion alliances in network systems from a game theoretical perspective [9].

As such, comprehending the multidimensional space of attributes (feature space) and the boundaries that differentiate various classes of network traffic within the feature space (decision boundaries) in commercial WAFs is critical for augmenting the efficacy of security systems for web applications. In this paper, we introduce a novel approach that employs maximum entropy in the mean to obtain insights into the feature space and decision boundaries of commercial WAFs. Our methodology enables defenders to reveal the underlying distribution of request features classified as malicious and benign by commercial WAFs, thereby bolstering their ability to defend against cyber-attacks.

The manuscript is organized as follows. First, to gain some perspective about the nature of the problem, in Section 2, we describe the data collection process. With this in mind, we explain the method of maximum entropy in Section 3. There we extend the results in [10] to cover the current augmented problem. In Section 4, we describe the results of the implementation of the maxentropic procedure. In Section 5, we present our concluding remarks.

## 2. Data Collection

In this study, we utilized the CSE-CIC-IDS2018 dataset for our analysis. This dataset is a publicly available collection of network traffic captures, which were recorded in a controlled environment using a custom-designed setup [11]. The dataset contains both benign and malicious traffic and is specifically designed to develop and evaluate network intrusion detection systems.

The CSE-CIC-IDS2018 is a dataset consisting of dynamically generated records for benign and malign events within a computer network [11].

The benign events are created by fitting a distribution to data collected from real users and subsequently drawing from the distribution using multiple machine learning and statistical analysis techniques.

The malign events were created by automated agents attacking the network under seven different scenarios described in the Appendix A in Table A1.

The variables we chose were the requests over ports and the attack scenarios described in Table A1. In a real-world network, each port can be directly associated with a computer application; thus, the information revealed by the joint distribution of attacks to each port provides defendants with a quantitative measurement of the attackers’ application target and behavior within the network. Additionally, the data required to find the joint distribution can be collected from the defendants’ firewall and monitoring system, which makes it accessible, and the results in the paper are appropriate for any cybersecurity team.

To process the dataset, we first limited the total number of records used to 80% and computed the frequency of port usage and requests for each class of traffic. The dataset included 14 classes of malicious requests and benign requests. Using this information, we then calculated the joint distribution of port usages and requests of each traffic class. Furthermore, we computed the individual probability of each port and traffic class under analysis. To understand the feature space and decision boundaries of commercial WAFs, we employed a novel approach using maximum entropy in the mean (MEM). MEM is a statistical method that allows us to estimate the underlying probability distribution of a set of features based on a sample of observations when only their probability is known. The data can, therefore, be explained with the following two variables, *X* and *Y*, in the following way:

- X: Traffic type, frequency of connections belonging to a: ’Benign’, ’Infilteration’, ’Bot’, ’Brute Force -Web’, ’Brute Force -XSS’, ’SQL Injection’, ’DoS attacks-GoldenEye’, ’DoS attacks-Slowloris’, ’DDoS attacks-LOIC-HTTP’, ’DDOS attack-HOIC’, ’DDOS attack-LOIC-UDP’, ’FTP-BruteForce’, ’SSH-Bruteforce’, ’DoS attacks-Hulk’, ’DoS attacks-SlowHTTPTest’. We shall label each of these 15 ports and from 1 to 15, respectively.
- Y: traffic behavior, frequency of port usage “0” “135” “137’ ’139” “21” “22” “3128” “3389” “443” “445” “53” “5355” “67” “80” “8080”. Those ports were selected since they represent more than 80% of web network traffic. We shall label each of these 15 ports from 1 to 15, respectively.

Additionally, the reason for selecting ports as the focal point of analysis is that they characterize network traffic with the most information; thus, each port is associated with a specific process or service, and it has been shown that the analysis of network traffic can provide the specific insight needed to develop novel approaches to collect, classify, and eventually, mitigate malware [12].

## 3. Statement of the Inverse Problem and Its Solution by MEM

The mathematical problem to be solved consists of determining a 15×15 matrix from the imprecise knowledge of the row and column sums. Let us write $p_{i,j}=P\left(X=x_{i},Y=y_{j}\right)$, where 1≤i≤15 and 1≤j≤15, and the values of *X* and *Y* are described above, in Section 2. So, to begin with, if there were no measurement errors, the problem to solve consists of determining a joint distribution from its marginals; that is:

(1) $$\begin{array}{cc} \mathrm{Determine}\;a\;\mathrm{joint}\;\mathrm{distribution} & \;\;\;\;p_{i,j}\;\;\;\mathrm{such}\;\mathrm{that}\\ 0\leq p_{i,j}\leq 1\;\;\;\mathrm{and} & \;\;\;\;\sum_{i=1,j=1}^{\left(15,15\right)}p_{i,j}.=1\\ \sum_{j=1}^{15}p_{i,j}:=p_{i}\;\;\;\;i=1,\ldots ,15\;\; & \;\;\sum_{i=1}^{15}p_{i,j}:=q_{i}\;\;\;\;j=1,\ldots ,15. \end{array}$$

To write this in a standard form, it is convenient to vectorize this problem. For that, we list the components of $p_{i,j}$ lexicographically into a vector x of N=15×15=225 components. Similarly, the row and column sums will be vectorized into a data vector d of dimension K=15+15=30, defined by

(2) $$d=\left(\begin{array}{c} p\\ q \end{array}\right)$$

The 30 row and column sum constraints upon $p_{i,j},$ will be vectorized into a 30×225 matrix C, the specification of which is shown in the Appendix A. Also, let u denote the 225-vector with all of its components equal to 1. As usual, we think of vectors as column vectors, and the superscript “${}^{t}$” denotes the transposition of the indicated object.

With these notations, after vectorization problem (1) becomes:

(3) $$\begin{array}{cc} \mathrm{Determine}\;a\;\mathrm{vector} & \;\;\;\;x\;\;\;\mathrm{such}\;\mathrm{that}\\ 0\leq x_{k}\leq 1\;\;\;\mathrm{and} & \;\;\;\;u^{t}x=1\\ Cx= & d. \end{array}$$

Observe that the constraint matrix can be augmented by adding $u^{t}$ as a first row to bC, so that the constraint $u^{r}x=1$ does not appear as an extra constraint. An important remark is the following. When two data vectors, p and q, are probabilities, then we do not have to impose this constraint. But when the marginals are not proportions; that is, when each of them does not add up to 1, we still may want to impose $u^{r}x=1.$ In this case, we augment the constraint matrix as follows:

Let us put

(4) $$A_{0}=\left(\begin{array}{c} u^{t}\\ C \end{array}\right)$$

The situation just described may occur when the data vector is known up to an observational error. In this case, besides augmenting the constraint matrix, the statement of problem (3) has to be modified because now the range of the matrix C, or that of $A_{0}$, may not contain the data point. Apart from redefining the constraint matrix, as in (4), we replace (3) with the following problem:

(5) $$\begin{array}{cc} \mathrm{Determine}\;a\;\mathrm{vector}\;\;\;\;x\in R^{225}\; & \;\mathrm{and}\;\;\;e\in {\left[-\delta ,\delta \right]}^{30}\;\;\;\mathrm{such}\;\mathrm{that}\\ 0\leq x_{k}\leq 1\;\;\;\mathrm{and}\;\;\; & \;\;\;A_{0}x+e=d. \end{array}$$

This is equivalent to regularizing the problem because it permits the solution to deviate from satisfying the constraints. The role of e is to absorb the error. We choose ${\left[-\delta ,\delta \right]}^{30}$ as the range of e, due to computational simplicity. The order of magnitude of the parameter δ is determined by a statistical analysis of the data. So, (5) is a typical ill-posed, linear problem with convex constraints.

In short, we use N=225 and K=30. To allow for the possibility of constraints upon the entries of some cells, we suppose that $x_{j}\in \left[a_{j},b_{j}\right]$ with $0\leq a_{j}<b_{j}\leq 1$ for j=1,…,N. This may occur when the probability and severity of a given type of attack lies within a known range. To estimate errors of measurement as part of the solution of the problem, we augment (5) as follows. We put

A=[A0IK],z=xeK=∏j=1N[aj,bj]×[−d,d]K.

A=[A0IK],z=xeK=∏j=1N[aj,bj]×[−d,d]K.

We shall use M=N+K=255. With these notations, the extension of the problem of reconstructing a joint probability from its marginals, when there is an error of measurement in the data, can be stated as follows:

(6) $$\begin{array}{cc} \mathrm{Determine}\;a\;\mathrm{vector} & \;\;\;z\in K\;\;\;\;\mathrm{such}\;\mathrm{that}\\ Az & =d. \end{array}$$

The method of maximum entropy in the mean (MEM) transforms the constrained ill-posed problem into an unconstrained, non-linear, convex optimization problem. Instead of solving (6), we search for a probability *P* on (K,F), where F denotes the class of Borel measurable subsets of K, such that

(7) $$z=AE_{P}\left[Z\right]=d.$$

This is the essence of the method of maximum entropy in the mean: To transform solving a linear ill-posed problem, subject to convex constraints, into a problem of searching for a probability measure on the space of constraints, such that the average of the coordinates with respect to the probability satisfies the constraint. Since the measure is concentrated within the constraint set, and the average over a convex set remains within the set, the convexity constraint is inherently satisfied.

Let Z:K→K be the coordinate (identity) mapping. This is a standard generalized moment problem. To determine *P* in this setup, it is easier to start from a reference measure Q, which we choose to define as

(8) $$dQ\left(\zeta \right)=\prod_{j=1}^{N}\left(\delta_{a_{j}}\left(d\xi_{j}\right)+\delta_{b_{j}}\left(d\xi_{j}\right)\right)\prod_{j=1}^{K}\left(\delta_{-d}\left(d\eta_{j}\right)+\delta_{d}\left(d\eta_{j}\right)\right).$$

This is chosen because any interior point in an interval can be written as a convex combination of the endpoints. Any probability *P* that is absolutely continuous with respect to *Q* (written P<<Q) has the form

(9) $$dQ\left(\zeta \right)=\prod_{j=1}^{N}\left(p_{j}\delta_{a_{j}}\left(d\xi_{j}\right)+q_{j}\delta_{b_{j}}\left(d\xi_{j}\right)\right)\prod_{i=1}^{K}\left(\alpha_{i}\delta_{-d}\left(d\eta_{i}\right)+\beta_{i}(\delta_{d}\left(d\eta_{i}\right)\right).$$

where the coefficients satisfy

$$p_{j}+q_{j}=1,\;\;\;j=1,\ldots ,N,\;\;\;\mathrm{and}\;\;\;\alpha_{i}+\beta_{i}=1,\;\;\;i=1,\ldots ,K.$$

When determining the coefficients $p_{j},\alpha_{i}$, the notion of entropy comes in. We will leave this as an exercise for the reader, to verify that the expected values of $\xi_{j}$ and $\eta_{i}$ are given by

(10) $$x_{j}=a_{j}p_{j}+b_{j}\left(1-p_{j}\right)\;\;\;\;\mathrm{and}\;\;\;\;e_{i}=-d\alpha_{i}+d\left(1-\alpha_{i}\right).$$

The class of probabilities that satisfies (7) is a convex se. When it comes to locating a point within a convex set, one effective approach is to maximize a convex function defined over that set. We define the entropy of *P* relative to *Q* by

(11) $$S_{Q}\left(P\right)=-\left(\sum_{j=1}^{N}p_{j}\ln p_{j}+\left(1-p_{j}\right)\ln\left(1-p_{j}\right)+\sum_{i=1}^{K}\alpha_{i}\ln\alpha_{i}+\left(1-\alpha_{i}\right)\ln\left(1-\alpha_{i}\right)\right).$$

With all this, we replace (6) with

(12) $$\begin{array}{cc} \mathrm{Determine}\;\;\;P< & <Q\;\;\;\mathrm{that}\;\mathrm{maximizes}\\ S_{Q}\left(P\right)=-(\sum_{j=1}^{N}p_{j}\ln p_{j}+\left(1-p_{j}\right)\ln\left(1-p_{j}\right) & +\sum_{i=1}^{K}\alpha_{i}\ln\alpha_{i}+\left(1-\alpha_{i}\right)\ln\left(1-\alpha_{i}\right)).\\ \mathrm{Subject}\;\mathrm{to}\;\;\;z & =AE_{P}\left[Z\right]=d. \end{array}$$

This clarifies the comment made immediately after (7). Once the probability in (12) is found, then $x=E_{P}\left[X\right]$ automatically satisfies the convexity constraints and equation Ax=bd. This problem is easy to solve using Lagrange multipliers. Going through the routine, Equation (10) yields:

(13) $$x_{j}^{*}=\frac{a_{j}e^{-a_{j}{\left(A_{0}^{t}\lambda^{*}\right)}_{j}}+b_{j}e^{-{\left(b_{j}A_{0}^{t}\lambda^{*}\right)}_{j}}}{e^{-a_{j}{\left(A_{0}^{t}\lambda^{*}\right)}_{j}}+e^{-b_{j}{\left(A_{0}^{t}\lambda \right)}_{j}^{*}}}\;\;\;\;e_{i}=\frac{-de^{d\lambda_{i}^{*}}+de^{-d\lambda_{i}}}{e^{d\lambda_{i}^{*}}+e^{-d\lambda_{i}^{*}}}.$$

When there is no prior information on the content of the cells, all values become $a_{j}=0$ and $b_{j}=1$, and (13) becomes

(14) $$x_{j}^{*}=\frac{e^{-{\left(b_{j}A_{0}^{t}\lambda^{*}\right)}_{j}}}{1+e^{-b_{j}{\left(A_{0}^{t}\lambda \right)}_{j}^{*}}}\;\;\;\;e_{i}=\frac{-de^{d\lambda_{i}^{*}}+de^{-d\lambda_{i}}}{e^{d\lambda_{i}^{*}}+e^{-d\lambda_{i}^{*}}}.$$

The next step is to elucidate how to obtain the optimal Lagrange multiplier $\lambda^{*}\in R^{K}.$ This is achieved by minimizing the convex function

(15) $$\Sigma \left(\lambda ,d\right)=\sum_{j=1}^{N}\ln\left(e^{-a_{j}{\left(A_{0}^{t}\lambda^{*}\right)}_{j}}+e^{-b_{j}{\left(A_{0}^{t}\lambda \right)}_{j}^{*}}\right)+\sum_{i=1}^{K}\ln\left(e^{d\lambda_{i}^{*}}+e^{-d\lambda_{i}^{*}}\right)+\langle \lambda ,d\rangle .$$

This is a strictly convex function that has a minimizer in $R^{K}$, as long as the datum d lies in the range of $A_{0}.$ It is easy to verify that the first-order condition for $\lambda^{*}$ to be a minimizer of Σ(λ,d) is equivalent to ensuring that $x^{*}$, as given in (13) or (14), satisfies (7).

## 4. Results

### 4.1. Simulation Exercise

In the following section, we perform a small toy example to test our method and see how well the reconstruction of a bivariate probability distribution is of two random variables, *X* and *Y*, which can both take the values of 1,2, and 3 (see Table 1).

We can easily compute the marginal distribution for *X* with probability vector p=(0.30,0.46,0.24), and for *Y*, with probability vector q=(0.19,0.47,0.34), respectively. We use the algorithm from [13], which yields the following Table 2 of the reconstructed probability distribution for δ=0, with a few converging steps:

We were able to reconstruct—to a very close level of accuracy—the theoretical joint probability distribution without errors. If we add an error of d=0.005, then we obtain the following reconstructed probability distribution (see Table 3):

### 4.2. Real Data Application

Here, we apply the method developed in Section 3 to obtain the joint probabilities that fit the data. We compute the distributions for *X* and *Y* from the data, and obtain the following probability vectors:

- $p=(1.791\times 10^{-2};1.109\times 10^{-3};1.723\times 10^{-3};2.895\times 10^{-4};2.509\times 10^{-2}$; $1.485\times 10^{-2};2.107\times 10^{-3};1.379\times 10^{-1};1.523\times 10^{-1};5.349\times 10^{-2};3.060\times 10^{-1}$; $3.817\times 10^{-3};$$1.018\times 10^{-3};2.612\times 10^{-1};2.121\times 10^{-2})$
- $q=(7.976\times 10^{-1};8.399\times 10^{-3};2.118\times 10^{-2};3.637\times 10^{-5};1.698\times 10^{-5};6.538\times 10^{-6}$; $3.119\times 10^{-3};8.259\times 10^{-4};4.330\times 10^{-2};5.155\times 10^{-2};1.300\times 10^{-4};1.453\times 10^{-2}$; $1.410\times 10^{-2};3.471\times 10^{-2};1.051\times 10^{-2})$

Considering the distribution vectors, it is crucial to take into account the high numerical resolution and the extensive dataset comprising over 13 million data points. This scale of data allows us to observe events with probabilities as low as $1\times 10^{-5}$, even if they are considered unlikely.

Finally, we minimized the entropy using the r package implemented by [13] and found the following joint distribution for both *X* and *Y*, where the gradient was equal to $4.618528\times 10^{-7}$. The results are presented in Table 4. This table contains the main computational results of this work. It contains the joint probabilities of the type of connections and the frequency of connections to the network.

## 5. Discussion

In conclusion, this paper presented a novel methodology that employs maximum entropy in the mean to gain insights into the feature space and decision boundaries of commercial web application firewalls (WAFs). This approach enables defenders to uncover the joint underlying distribution of requests classified as malicious and benign by commercial WAFs, thereby enhancing their ability to protect against cyber-attacks, when taking into account the dependence between the types of attacks.

The bivariate density reconstructed in Table 4 enabled us to evaluate the most probable future attacks, along with conditional probabilities of an attack occurring based on factors such as internet traffic volume or any other relevant measurable variable that we can use for conditioning.

We demonstrate that our methodology could reconstruct a joint distribution of different traffic types (benign and malicious) and the port usage for each class with an error of less than $1\times 10^{-4}$ at the gradient level. This level of accuracy allows for the precise identification of the amount of incoming malicious traffic for each port. Furthermore, since our methodology can be employed to reconstruct the joint distribution of any two variables, it can be utilized to pinpoint any measurable feature for malicious traffic.

The results presented in this paper not only emphasize the importance of understanding the feature space and decision boundaries in commercial WAFs, but also showcase the effectiveness of the proposed methodology in addressing the critical aspect, such as the dependence of different types of attacks in cyber security. By providing a robust and versatile tool for analyzing and interpreting the characteristics of malicious traffic, this research significantly contributes to the ongoing efforts in the cybersecurity community in regard to developing and implementing innovative solutions for safeguarding individuals and organizations in the constantly evolving digital landscape.

As part of future work, we recommend further exploring the applicability of our methodology to other types of security systems and exploring its potential for integration into existing intrusion detection frameworks. Additionally, ongoing research on the identification and analysis of measurable features for malicious traffic will help to refine and expand the capabilities of our approach, ultimately contributing to the development of more comprehensive and effective strategies for defending against cyber threats.

## Figures and Tables

**Table 1:** True joint probability distribution.

	Y=1	Y=2	Y=3
X=1	0.09	0.14	0.07
X=2	0.07	0.23	0.16
X=3	0.03	0.10	0.11

**Table 2:** Reconstructed probability distribution without errors.

	Y=1	Y=2	Y=3
X=1	0.06	0.14	0.10
X=2	0.09	0.21	0.16
X=3	0.04	0.11	0.08

**Table 3:** Reconstructed probability distribution with error d=0.005.

	Y=1	Y=2	Y=3
X=1	0.06	0.14	0.10
X=2	0.09	0.21	0.16
X=3	0.04	0.11	0.08

**Table 4:** Reconstructed probability distribution from data.

	1	2	3	4	5	6	7	8	9	10	11	12	13	14	15
1	1.77 × 10${}^{-2}$	1.69 × 10${}^{-4}$	1.93 × 10${}^{-5}$	1.57 × 10${}^{-6}$	0.00	0.00	0.00	0.00	0.00	0.00	0.00	0.00	0.00	0.00	0.00
2	9.14 × 10${}^{-4}$	1.93 × 10${}^{-4}$	0.00	0.00	0.00	0.00	0.00	0.00	0.00	0.00	0.00	0.00	0.00	0.00	0.00
3	1.70 × 10${}^{-3}$	2.00 × 10${}^{-5}$	0.00	0.00	0.00	0.00	0.00	0.00	0.00	0.00	0.00	0.00	0.00	0.00	0.00
4	2.21 × 10${}^{-4}$	6.77 × 10${}^{-5}$	0.00	0.00	0.00	0.00	0.00	0.00	0.00	0.00	0.00	0.00	0.00	0.00	0.00
5	3.93 × 10${}^{-5}$	2.63 × 10${}^{-6}$	0.00	0.00	0.00	0.00	0.00	0.00	0.00	0.00	0.00	1.45 × 10${}^{-2}$	2.25 × 10${}^{-6}$	0.00	1.05 × 10${}^{-2}$
6	6.88 × 10${}^{-4}$	7.05 × 10${}^{-5}$	0.00	7.51 × 10${}^{-8}$	0.00	0.00	0.00	0.00	0.00	0.00	0.00	0.00	1.40 × 10${}^{-2}$	0.00	0.00
7	2.10 × 10${}^{-3}$	2.10 × 10${}^{-6}$	0.00	0.00	0.00	0.00	0.00	0.00	0.00	0.00	0.00	0.00	0.00	0.00	0.00
8	1.36 × 10${}^{-1}$	1.02 × 10${}^{-3}$	0.00	0.00	0.00	0.00	0.00	0.00	0.00	0.00	0.00	0.00	0.00	0.00	0.00
9	1.50 × 10${}^{-1}$	2.19 × 10${}^{-3}$	0.00	0.00	0.00	0.00	0.00	0.00	0.00	0.00	0.00	0.00	0.00	0.00	0.00
10	5.30 × 10${}^{-2}$	4.10 × 10${}^{-4}$	0.00	0.00	0.00	0.00	0.00	0.00	0.00	0.00	0.00	0.00	0.00	0.00	0.00
11	3.02 × 10${}^{-1}$	3.78 × 10${}^{-3}$	0.00	0.00	0.00	0.00	0.00	0.00	0.00	0.00	0.00	0.00	0.00	0.00	0.00
12	3.80 × 10${}^{-3}$	8.49 × 10${}^{-6}$	0.00	0.00	0.00	0.00	0.00	0.00	0.00	0.00	0.00	0.00	0.00	0.00	0.00
13	1.00 × 10${}^{-3}$	8.64 × 10${}^{-6}$	0.00	1.50 × 10${}^{-7}$	7.51 × 10${}^{-8}$	0.00	0.00	0.00	0.00	0.00	0.00	0.00	0.00	0.00	0.00
14	1.27 × 10${}^{-1}$	4.37 × 10${}^{-4}$	0.00	3.45 × 10${}^{-5}$	1.69 × 10${}^{-5}$	6.53 × 10${}^{-6}$	3.11 × 10${}^{-3}$	8.25 × 10${}^{-4}$	4.32 × 10${}^{-2}$	5.15 × 10${}^{-2}$	1.30 × 10${}^{-4}$	0.00	0.00	3.47 × 10${}^{-2}$	0.00
15	4.33 × 10${}^{-5}$	2.70 × 10${}^{-6}$	2.11 × 10${}^{-2}$	0.00	0.00	0.00	0.00	0.00	0.00	0.00	0.00	0.00	0.00	0.00	0.00

## Data Availability

Not applicable.

## Appendix A

### The Vectorized Form of the Constraint Matrix

For numerical processing, we already mentioned that it is convenient to cast the 15×15 joint probability matrix as a 225 vector $x\in R^{225},$ subject to positivity and other constraints. Thus, it is also necessary to rewrite the constraints as linear or convex constraints upon x. For that, we denote by 1 (resp. 0) the vector in $R^{15}$ with all entries equal to 1 (resp. equal to 0), let $1^{t}$ and $0^{t}$ be their transposes, and let I denote the 15×15 identity matrix and form the 30×225 matrix:

(A1) $$\;C=\left(\begin{array}{cccc} 1^{t} & 0^{t} & \ldots & 0^{t}\\ 0^{t} & 1^{t} & \ldots & 0^{t}\\ \vdots & \vdots & \ldots & \vdots \\ 0^{t} & 0^{t} & \ldots & 1^{t}\\ I & I & \ldots & I \end{array}\right)$$

From now on, the remainder continues as described in Section 3.

**Table A1:** Table with descriptions of attacks.

Scenario	Description
Brute-force attack	A set of agents attempts to connect via SSH or FTP to machines within the network by guessing the system’s password.
Heartbleed attack	Heartbleed is a vulnerability found in the OpenSSL library, which allows an attacker to leak memory data from a system. Some vulnerable machines were added to the network and the heartleech program was used to exploit them.
Botnet	Botnets are computers infected with malicious software and controlled as a group without the owners’ knowledge. Some computers within the network were infected with Zeus and other types of Trojans used to create botnets.
Denial-of-Service (DoS)	This attack seeks to shut down a machine or network, making it inaccessible to its intended users. An agent was used to attack the systems within the network using the Slow Loris variant of this attack.
Distributed Denial-of-Service (DDoS)	DDoS is a variant of the DoS attack, where multiple agents (usually a botnet) are used to overwhelm the target with a huge amount of requests. An agent was tasked to perform stress tests on the services and simulate a DDoS.
Web Attacks	Web attacks aim to exploit vulnerable web applications (such as websites). Some computers within the network ran a vulnerable PHP/MySQL web application and an agent was used to automatically exploit the vulnerabilities.
Infiltration of the network from inside	This scenario simulates the actions of an attacker who has gained control of one of the computers from within the network and uses Nmap to perform an IP sweep, full port scan, and service enumerations.
